# Diversity and Faunal Composition of Coleoptera in the Gansu Heihe Provincial Nature Reserve, China

**DOI:** 10.3390/insects17070700

**Published:** 2026-07-06

**Authors:** Jia Qi, Kang Chang, Jianhui Wang, Miao Li, Lulin Li, Xiaoxiao Chen, Suqin Shang, Youssef Dewer

**Affiliations:** 1 Biocontrol Engineering Laboratory of Crop Diseases and Pests of Gansu Province, College of Plant Protection, Gansu Agricultural University, Lanzhou 730070, China; 18794180891@163.com (J.Q.); 15294346907@163.com (K.C.); 15095553575@163.com (J.W.); 19931810726@163.com (M.L.); chenxx@gsau.edu.cn (X.C.); 2 School of Environmental Engineering, Gansu Forestry Voctech University, Tianshui 741000, China; 3 Gansu Heihe Provincial Nature Reserve Administration, Tianshui 741020, China; 18919216935@163.com; 4 Department of Phytotoxicity Research, Central Agricultural Pesticide Laboratory, Agricultural Research Center, 7 Nadi El-Seid Street, Dokki, Giza 12618, Egypt

**Keywords:** Coleoptera, biodiversity, faunal composition, Heihe Provincial Nature Reserve, Gansu

## Abstract

**Background**: The Gansu Heihe Provincial Nature Reserve harbors rich plant and animal resources, yet its coleopteran fauna has remained taxonomically and ecologically uncharacterized. To address this gap, we conducted the first systematic survey of beetle diversity across four representative transects—Youcaigou (I), Zhugou (II), Loufangya (III), and Sigou (IV)—and assessed variation in community structure using the Shannon–Wiener diversity index, Margalef richness index, Berger–Parker dominance index, Pielou evenness index, and Jaccard similarity coefficient. **Results**: A total of 131 species belonging to 108 genera and 22 families were identified, establishing a comprehensive baseline of beetle diversity for the reserve. Cerambycidae was the most species-rich family (27 species), while Lampyridae, Staphylinidae, and Anobiidae were each represented by only a single species, reflecting marked unevenness in family-level composition. Diversity and community structure differed substantially among transects: Transect II showed the highest Shannon–Wiener diversity index and Pielou evenness, Transect III the lowest diversity, and Transect IV the greatest dominance by a few species, while species richness declined in the order I > II > III > IV. Zoogeographic analysis revealed that widely distributed (Cosmopolitan) species predominated (83 species; 63.36%), with Palaearctic (21 species; 16.03%) and Oriental (27 species; 20.61%) elements also well represented, underscoring the reserve’s position at a key biogeographic transition zone. Within China, North China faunal elements were most prevalent (75.94%), with the North China–Northeast China distribution group alone accounting for 54.20% of species, whereas nationally widespread species comprised only 9.16%. **Conclusions**: This study provides the first systematic record of coleopteran diversity in the Gansu Heihe Provincial Nature Reserve, enriching the region’s biodiversity inventory and offering reference data for future insect monitoring and forest conservation. Further multi-seasonal, method-diversified surveys are needed to achieve a complete faunal inventory.

## Simple Summary

Beetles (Coleoptera) are highly diverse and ecologically important, yet the beetle fauna of the Gansu Heihe Provincial Nature Reserve in northwestern China has not been studied. We conducted the first systematic survey, sampling four habitat transects monthly over one season using multiple collection methods. We recorded 131 species from 108 genera and 22 families, with longhorn beetles (Cerambycidae) being the most species-rich group. Diversity was highest where vegetation cover was good and human disturbance was low. Most species were widely distributed across China, reflecting the reserve’s position at a major biogeographic transition zone. This preliminary inventory provides baseline data for future surveys and conservation planning.

## 1. Introduction

As one of the most species-rich and ecologically diverse groups within Insecta, Coleoptera plays a vital role in terrestrial ecosystems and has attracted substantial scientific interest [1,2,3]. Beetles hold significant theoretical and applied value in biodiversity conservation [4], beneficial insect utilization [5], faunal surveys [6], zoo geography [7,8], and agricultural pest management [9,10,11]. Their diversity extends beyond species richness to genetic, trait, and functional dimensions [12,13,14]. Because beetles are highly sensitive to environmental change [15], they serve as effective bioindicators [16,17,18], with shifts in abundance and composition reflecting ecosystem health [19]. Consequently [20], patterns of coleopteran diversity and distribution have become central to biodiversity research [21,22].

The Heihe Nature Reserve, Gansu Province, lies in the western Qinling Mountains, bordering Tongtianhe National Forest Park (Shaanxi) to the northeast and forest farms of the Xiaolongshan Forestry Protection Center to the northwest and southwest, placing it at the junction of major forest systems with notable geographical significance. As a forest-ecosystem-type reserve, it protects natural secondary forests, subtropical landscapes, and habitats of rare and endangered species, playing a key role in regional ecological stability. Its hydrological network includes first-order tributaries of the Jialing River, with the Heihe River (~200 km; drainage area ~5000 km^2^) serving as the main stream, supplying water, regulating climate, and supporting diverse organisms, with waterfalls and streams further enhancing local biodiversity [23,24].

Insect distribution is strongly shaped by regional ecological conditions [25], and vegetation type profoundly influences habitat selection and reproduction [26]. Situated at the transition between subtropical and warm-temperate climates, the reserve hosts forest ecosystems of exceptional representativeness [27,28], with vegetation spanning coniferous forests, broadleaf forests, shrublands, and grasslands. To date, 994 vascular plant species (111 families, 461 genera) have been recorded, including 40 fern, 11 gymnosperm, and 943 angiosperm species [29]. Encompassing nearly the full vegetation spectrum of the Qinling region with well-preserved primary vegetation, the reserve provides an invaluable platform for in situ conservation and biogeographical research [30].

Coevolution between insects and plants has produced intricate mutualisms [31]: insects feed on and pollinate plants, while plants provide habitat and resources in return [32]. As key components of food webs, insects contribute to energy flow, nutrient cycling, and soil fertility [33], making insect diversity a sensitive indicator of ecosystem health [34,35]. With its rich biodiversity and intact ecological structure, the Heihe Nature Reserve functions as an ecological security barrier in the western Qinling Mountains, supporting water conservation, soil protection, and climate regulation, an ecological asset of considerable importance to northwestern China.

Despite this significance, insect biodiversity in the reserve remains poorly characterized, and no published study has examined coleopteran community structure in relation to habitat variation here. We therefore established four sampling transects for systematic field survey, employing a combination of sweep-netting, hand-collecting, knockdown sampling, Malaise trapping, pitfall trapping, and light trapping. However, no single combination of methods can fully capture the diversity of a hyperdiverse order such as Coleoptera; cryptic or soil-dwelling families—notably Staphylinidae, one of the largest beetle families worldwide (~65,000 species globally, >6000 in China)—are likely underrepresented by these methods. This limitation should be considered when interpreting family- and genus-level patterns reported here.

Accordingly, rather than addressing broader questions of faunal origin or long-term conservation strategy, this study aims to characterize the taxonomic composition of coleopteran assemblages across the reserve’s major habitats and document species distribution patterns among transects, providing baseline data to support future ecological monitoring and conservation planning.

## 2. Materials and Methods

### 2.1. Study Area

The Heihe Provincial Nature Reserve is located in southeastern Gansu Province, within the western Qinling Mountains, in the upper reaches of the Jialing River. The reserve lies between 106°30′11″–106°34′25″ E and 34°8′39″–34°15′25″ N. This region constitutes a major biogeographical transition zone between the Palaearctic and Oriental realms, lending the reserve considerable zoogeographical significance. The terrain is topographically complex, characterized by pronounced variation in elevation and relief. Combined with a mild climate and relatively abundant rainfall, this topographic heterogeneity has shaped a distinctive landscape that provides ecologically diverse habitats supporting Coleoptera and a wide range of other organisms.

### 2.2. Survey and Specimen Collection

All coleopteran specimens were collected from the Gansu Heihe Provincial Nature Reserve between June and September 2024. Surveys were conducted monthly, each lasting 5–7 days, and covering all sample transects. Fieldwork was carried out on sunny days between 10:00 and 17:00, with specimens collected within a 2 m belt on either side of each transect line. Given the ecological characteristics of the reserve and the behavioral diversity of beetles, multiple complementary collection methods were employed, including sweep-netting, hand-collecting, knockdown sampling, Malaise trapping, pitfall trapping, and light trapping.

For beetles inhabiting foliage or branches, the knockdown method was used. A white plastic sheet or cloth was spread beneath the canopy of trees, shrubs, or tall-stemmed plants, and branches were gently tapped with a bamboo pole. Taking advantage of the beetles’ thanatosis (death-feigning behavior), individuals that fell onto the sheet were collected using forceps or an aspirator.

Light trapping was used to target phototactic beetles. On clear nights, light traps were set up in open areas or at forest edges, operated from 20:00 to 06:00 using a 450 W high-pressure mercury lamp paired with an insect-trapping tent.

Pitfall traps were used to capture ground-active beetles and consisted of disposable 500 mL plastic cups buried flush with the soil surface, each filled with a sugar-vinegar attractant solution (brown sugar: white vinegar: 75% ethanol: water = 1:4:1:16). Ten pitfall traps were deployed per sample transect, evenly spaced at approximately 50 m intervals along the transect line. Traps were set on the first day of each monthly survey and retrieved on the final day (5–7 days later), with the attractant solution replenished every 48 h. This baited pitfall design was chosen to enhance capture efficiency for ground-active Coleoptera; however, we acknowledge that a single array of ten traps per transect may underestimate true ground-beetle diversity, and future surveys should incorporate a greater number of traps distributed across the different biotope types present along each transect.

Following preliminary field sorting, all specimens were preserved in 75% ethanol and subsequently prepared as pinned specimens for laboratory-based species identification, following standard entomological preparation protocols (Egorov et al., 2024) [13].

The climate factor data, including temperature, humidity, total solar radiation, total rainfall, etc., used in this study were provided by government agencies, and can be found at the following URL (accessed on 26 January 2026): https://meteo.agrodigits.com/home/index [36].

### 2.3. Sample Line Design

**Figure 1:**
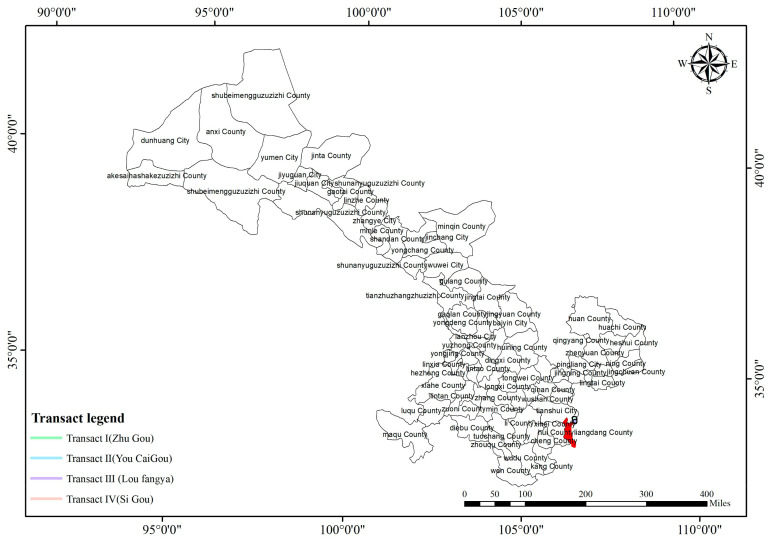
Location of the research area in the Heihe Provincial Nature Reserve, Gansu Province, China.

**Figure 2:**
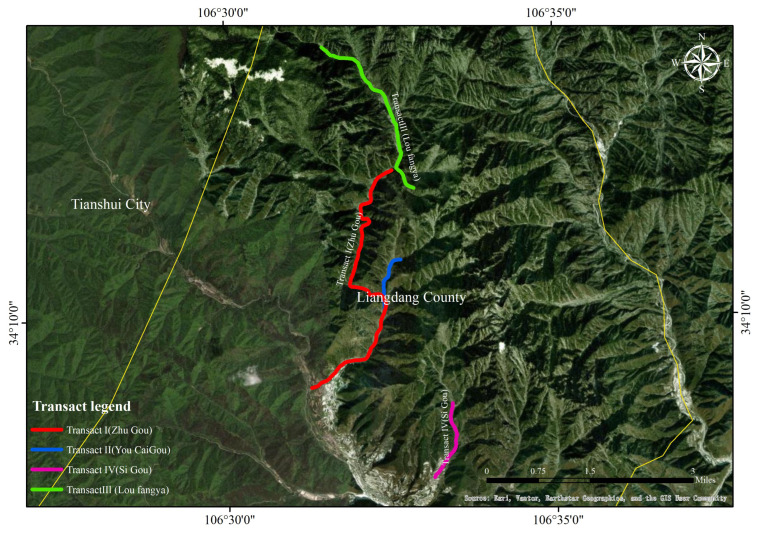
Location of the four sampling transects within the Heihe Provincial Nature Reserve, Gansu Province, China.

Based on dominant vegetation types and geographical features within the Gansu Heihe Provincial Nature Reserve, four sample transects were established (Figure 1 and Figure 2). Detailed information for each sample transect is provided in Table 1.

### 2.4. Data Analysis

To analyze the community structure across different sampling transects, α-diversity indices were calculated as described below [37].

The Shannon–Wiener diversity index (H′) was used to quantify species diversity, incorporating both species richness and relative abundance [38].

The Berger–Parker dominance index (D) was applied to determine the dominance of the most abundant species in the community [39].

The Pielou evenness index (J) measured the equitability of individuals across species [40,41].

The Margalef richness index (R) assessed species richness relative to the total number of individuals [42].

Shannon–Wiener diversity index (H′):

(1) $$P_{i}=N_{i}/N;H^{\prime }=-\sum P_{i}\ln P_{i}$$

where (pi) is the proportion of individuals belonging to the i-th species, (Ni) is the number of individuals of the i-th species, and (N) is the total number of individuals of all species.

Berger–Parker Dominance Index (D):

(2) $$D=N_{max}/N_{t}$$

where (Nmax) is the number of individuals of the most abundant species, and (Nt) is the total number of individuals.

Pielou Evenness Index (J):

(3) $$J=H^{\prime }(GS)/\ln S$$

where H′ is the Shannon–Wiener diversity index, and S is the total number of species.

Margalef richness index (R)

(4) R=(S−1)/lnN

where S in the number of species, and N is the total number of individuals.

Jaccard similarity coefficient was employed to evaluate species similarity between pairs of transects as follows [43]:

(5) I=c/(a+b+c)

where (a) and (b) are the number of species in transects A and B, respectively, and (c) is the number of species common to both transects. According to Jaccard’s principle, values close to 0 indicate extremely dissimilar communities, values between 0.25 and 0.50 indicate moderately dissimilar communities, values between 0.50 and 0.75 indicate moderately similar communities, and values above 0.75 indicate extremely similar communities.

## 3. Results

### 3.1. Community Composition of Coleoptera

A total of 1265 coleopteran specimens were collected from the four sampling transects in the Heihe Provincial Nature Reserve, Gansu Province in 2024 (see summary in Table 2). Specimens were identified using authoritative taxonomic literature, including Fauna Sinica, Economic Insect Fauna of China, Insect Fauna of the Qinling Mountains, supplemented by online resources such as the China Animal Subject Database and the National Animal Specimen Resource Database. The identification process classified the specimens into 131 species, 108 genera, and 22 families. Cerambycidae and Coccinellidae were the most species-rich. In contrast, Lampyridae, Staphylinidae, and Anobiidae were each represented by only a single species. The geographical affinities (fauna composition) of all specimens were determined using regional references such as Coleoptera Fauna of Ningxia, Insects of the Helan Mountains in Ningxia, and Insect Fauna of the Qinling Mountains.

At the genus level, *Lagria* contained the highest number of species (four). The genera *Holotrichia*, *Protaetia*, *Themus*, and *Harpalus* each included three species. Eleven genera comprised two species each: *Monochamus*, *Callidium*, *Melolontha*, *Lycostomus*, *Coccinella*, *Calvia*, *Cymindis*, *Diabrotica*, *Aulacophora*, *Cryptocephalus*, and *Agrilus*. The remaining 86 genera were monospecific, accounting for 84.31% of the total.

At the species level, *Epilachna plicata* was the most abundant, with 60 individuals, constituting the dominant species. Other abundant species included *Lagria nigricollis*, *Themus imperialis*, *Necrophorus halensis*, *Harmonia axyridis*, and *Aulacophora lewisii*, each represented by 40–50 specimens. Forty-six species, such as *Trichoferus campestris*, *Ischnostrangalis kubani*, and *Polyphylla laticollis*, were represented by a single individual and were classified as rare.

### 3.2. Faunal Distribution of Coleoptera

#### 3.2.1. Regional Characteristics in the World’s Zoogeographical Regions

**Figure 3:**
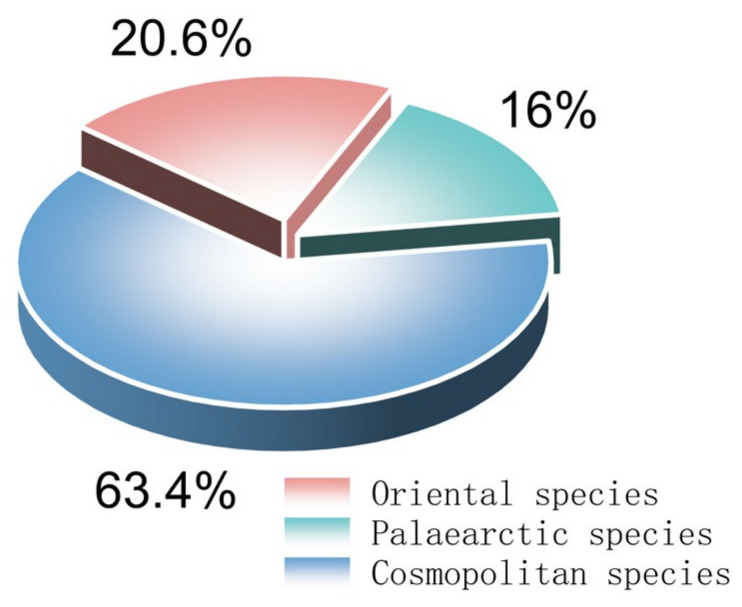
Proportional representation of Coleoptera species from the Gansu Heihe Provincial Nature Reserve within the world’s major zoogeographical regions (Cosmopolitan, Palaearctic, Oriental).

The global zoogeographical affinities of the 131 coleopteran species are shown in Figure 3. The fauna of the reserve comprises Cosmopolitan, Oriental, and Palaearctic elements. Cosmopolitan species were the most abundant, accounting for 63.4% of the total species and predominating in both richness and overall community composition.

#### 3.2.2. Regional Characteristics in the Chinese Zoogeographical Regions

Following Chinese zoogeographical divisions, the Palaearctic realm is subdivided into the Northeast, North China, Qinghai-Tibet, and Inner Mongolia-Xinjiang Regions, while the Oriental realm includes the Central China, Southwest China, and South China regions. The Heihe Provincial Nature Reserve is situated within the North China Region of the Palaearctic realm.

A total of 41 distinct distribution patterns were identified among the collected species (Table 3). Within these, 99 species (75.57%) occur in the North China Region. The combined North China-Northeast China distribution pattern contained the largest number of species, with 71 species (54.20%). Species with a nationwide distribution accounted for only 9.16% of the total.

### 3.3. Occurrence Dynamics of Coleoptera

**Figure 4:**
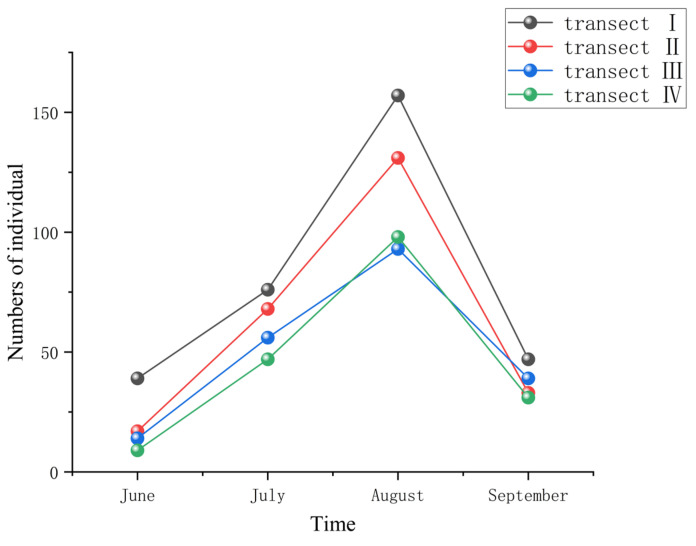
Seasonal variation in coleopteran abundance across the four sampling transects (I–IV) in the Gansu Heihe Provincial Nature Reserve, based on monthly collections from June to September 2024.

The abundance of coleopteran individuals across the four transects generally increased to a peak before declining over the sampling period, as shown in Figure 4. In June, Transect I recorded the highest species count, with 39 species. The overall peak in abundance across all transects occurred in August, with 157 individuals in Transect I, 131 in II, 93 in III, and 98 in IV, totaling 479 individuals. In August, collection represented 50% of the annual total, indicating that the primary activity period for Coleoptera in the reserve occurs during the summer months (June–August), with August being the peak.

### 3.4. Effects of Environmental Factors on Coleoptera Insect Diversity

**Figure 5:**
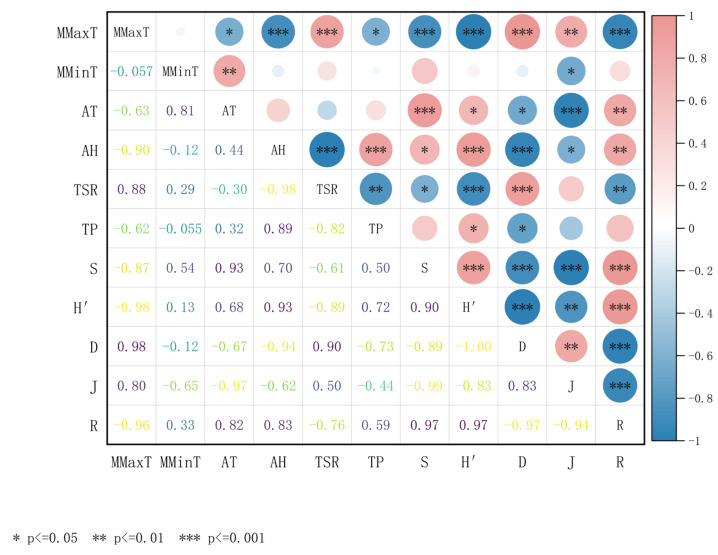
Correlation analysis between key environmental factors (temperature, humidity, total solar radiation, precipitation) and coleopteran community diversity indices (Shannon–Wiener H′, Pielou J, Berger–Parker D, Margalef R) for the Gansu Heihe Provincial Nature Reserve.

In this study, average temperature, average humidity, total solar radiation, total precipitation, monthly maximum temperature, and monthly minimum temperature were selected as environmental factors. The correlations between the Shannon–Wiener diversity index, Berger–Parker dominance index, Pielou evenness index, and Margalef abundance index and key environmental factors were analyzed. The results are shown in Figure 5.

Species richness exhibited a highly significant positive correlation with average temperature (r = 0.93), and also showed positive correlations with average humidity (r = 0.70) and monthly minimum temperature (r = 0.54), indicating that improved temperature conditions significantly promote species abundance. A weak positive correlation was observed with total precipitation (r = 0.32), suggesting that precipitation exerts a relatively limited stimulatory effect on species richness. Conversely, negative correlations were found with total solar radiation (r = −0.61) and monthly maximum temperature (r = −0.87), with the monthly maximum temperature exhibiting a particularly pronounced negative influence. This indicated that excessively high extreme temperatures may exert a suppressive effect on species richness.

The diversity index exhibited a highly significant positive correlation with average humidity (r = 0.93), and positive correlations with average temperature (r = 0.68) and total precipitation (r = 0.72), indicating that mild, humid thermohygric conditions contributes to enhancing community diversity. It exhibited a highly significant negative correlation with total solar radiation (r = −0.89) and monthly maximum temperature (r = −0.98), indicating that intense solar radiation and extremely high temperatures significantly reduce community diversity levels. The correlation with monthly minimum temperature approached zero (r = −0.13), indicating that low temperatures exert a relatively weak influence on the diversity index.

The species richness index exhibited a strong positive correlation with average temperature (r = 0.82) and average humidity (r = 0.83), indicating that synergistic improvements in thermal and hydrological conditions effectively enhance community species richness. It showed a weak positive correlation with monthly minimum temperature (r = 0.33) and an extremely weak positive correlation with total precipitation (r = 0.59), suggesting that these factors exert relatively limited promotional effects on species richness. It exhibited negative correlations with total solar radiation (r = −0.76) and monthly maximum temperature (r = −0.96), with the negative effect of monthly maximum temperature being particularly pronounced. This indicated that extreme heat is one of the key factors suppressing species richness.

The dominance index exhibited a highly significant positive correlation with total solar radiation (r = 0.90) and monthly maximum temperature (r = 0.98), indicating that environments characterized by intense radiation and high temperatures are more conducive to the most abundant species occupying ecological niches, thereby enhancing community dominance. It exhibited weak negative correlations with average temperature (r = −0.67) and total precipitation (r = −0.73), alongside a highly significant negative correlation with average humidity (r = −0.94), indicating that high-humidity environments significantly inhibit the competitive advantage of dominant species. The correlation with monthly minimum temperature approached zero (r = 0.12), suggesting negligible influence of low temperatures on dominance.

The evenness index exhibited a highly significant negative correlation with average temperature (r = −0.97) and a significant negative correlation with monthly minimum temperature (r = −0.65), indicating that rising temperatures significantly reduce species evenness within communities. Weak negative correlations were observed with mean humidity (r = −0.62) and total precipitation (r = −0.44), suggesting that these factors exert relatively limited effects on evenness. It exhibited an extremely weak positive correlation with total solar radiation (r = 0.50) and a positive correlation with monthly maximum temperature (r = 0.80), with the positive effect of monthly maximum temperature being particularly pronounced. This suggested that extreme high temperatures may, to a certain extent, promote the species abundance distribution.

Based on the above analysis, the comprehensive impact strength of various environmental factors on the diversity characteristics of Coleoptera insect communities, ranked from highest to lowest, is as follows: monthly maximum temperature (absolute range of correlation coefficients: 0.80–0.98) > average humidity (0.63–0.97) > average temperature (0.63–0.97) > total solar radiation (0.50–0.90) > total precipitation (0.32–0.73) > monthly minimum temperature (0.12–0.65). This finding indicates that monthly maximum temperature and average humidity are the key environmental factors regulating Coleoptera community diversity in this reserve, with excessively high temperatures or unsuitable humidity levels reducing community diversity, while appropriate temperature and humidity conditions help maintain and enhance community stability and complexity.

### 3.5. α-Diversity Across Transects

The *α*-diversity indices for the coleopteran communities in the four transect are presented in Table 4. The Shannon–Wiener diversity index was highest in Transect II (10.2239) and lowest in Transect III (10.0291). The Berger–Parker dominance index was highest in Transect IV (0.0865), with the dominance ranking order being IV > I > III > II. The Pielou evenness index was also highest in Transect II (0.8987). The Margalef richness index followed the order: Transect I > II > III > IV.

Overall, the patterns of species number, individual abundance, diversity, and richness were generally consistent. Transect II exhibited relatively higher diversity and evenness, suggesting greater vegetation variety and a more stable, favorable environment for Coleoptera.

### 3.6. Community Similarity Between Transects

The Jaccard similarity coefficients between transect pairs are shown in Table 5. The coefficients for Transects I–III and II–IV ranged from 0.25 to 0.50, indicating moderate dissimilarity. All other pairwise comparisons yielded coefficients between 0.50 and 0.75, indicating moderate similarity.

## 4. Discussion

This study provides the first systematic investigation of coleopteran fauna and diversity in the Heihe Provincial Nature Reserve, Gansu Province, recording a total of 131 species belonging to 108 genera and 22 families. At the genus level, monospecific genera were markedly predominant, comprising 86 of 108 genera (84.31%) represented by a single species each. While such a high proportion of monospecific genera might be interpreted as evidence of a faunal assemblage with complex biogeographic origins shaped by the reserve’s distinctive climate, vegetation, and topography, this interpretation warrants caution: the pattern may equally reflect inherent limitations of the sampling methods employed rather than a genuine biological signal. As a clear illustration, the family Staphylinidae—one of the most species-rich beetle families in China, with more than 6000 described species (Catalogue of Chinese Coleoptera, 2018) [44]—was represented by only a single specimen in this survey, underscoring the extent to which ground-dwelling and cryptic taxa remain underrepresented by the collection methods used here. Sampling effort was inevitably weighted towards manually accessible, canopy-associated, and wood-boring forms—reflected in the strong dominance of Cerambycidae—while sap-feeding, soil-dwelling, and nocturnal taxa were comparatively undersampled. The results presented here should therefore be regarded as a preliminary, methodologically constrained inventory rather than a complete characterization of the reserve’s coleopteran fauna. Future surveys incorporating window traps, canopy interception traps, rearing from woody substrates, and year-round pitfall arrays will be essential to more fully capture the beetle diversity of the reserve [45].

At the family level, Cerambycidae was the most species-rich group, comprising 27 species, whereas Lampyridae, Staphylinidae, and Anobiidae were each represented by only a single species—a disparity that, as noted above, likely reflects sampling bias toward visually conspicuous, diurnal, and wood-associated taxa rather than true differences in underlying family-level richness.

Considered jointly, species diversity and evenness were highest in Transect II, which was dominated by oak-broadleaf mixed forest across sunny slopes, the lower sections of semi-sunny slopes, and flat ridges. The relatively even abundance of species across taxonomic groups in this transect suggests a more balanced community structure compared to the other transects. This pattern is consistent with findings from studies on Xinglongshan white butterfly diversity [46] and Coleoptera diversity in central Inner Mongolia [47], both of which similarly link favorable vegetation conditions and moderate disturbance levels to elevated biodiversity.

Analysis of the Margalef richness index identified Transect I as having the highest species richness (14.9171), while Transect IV recorded the lowest (13.6006). Transect IV borders the Zhangjia Forest Farm and lies adjacent to village collective forests, and is consequently subject to frequent human activity and substantial disturbance [48,49]. This disturbance regime appears to have driven increased community dominance alongside reduced species richness, a pattern potentially attributable to vegetation homogenization, localized habitat degradation, or habitat fragmentation resulting from human activity [50,51,52]. More broadly, anthropogenic pressures such as excessive development, pollution, and habitat destruction are known to degrade beetle habitats, with corresponding declines in regional species diversity and abundance [15]. These findings underscore the importance of mitigating human disturbance to support the recovery of species diversity in Transect IV and comparably impacted areas of the reserve.

From a broader zoogeographic perspective, widespread species dominate the beetle fauna of the Heihe Nature Reserve, a pattern closely tied to the reserve’s distinctive geographical position. Located in the western Qinling Mountains along the boundary between the Palaearctic and Oriental realms, the reserve forms a classic biogeographic transition zone that facilitates the interpenetration of beetle taxa from both realms, producing a fauna characterized by mixed and intertwined biogeographic components. This pattern parallels the pronounced faunal overlap documented for Coleoptera in the Helan Mountains of Inner Mongolia [53], although the two regions differ meaningfully in habitat structure and climate: the Helan Mountains fauna reflects adaptation to a temperate, semi-arid environment, whereas the convergence of subtropical and temperate climatic influences in the Heihe Reserve appears to permit deeper penetration and overlap between beetle faunas of different biogeographic origin.

The Gansu Heihe Provincial Nature Reserve falls within the North China biogeographic region as defined in the *Biogeographic Rregions of China*. As a major geo-climatic boundary in eastern China, the Qinling Mountains constitute the core transitional zone separating the North China, Central China, and Mongolian regions, providing the biogeographical foundation for the reserve’s faunal diversity. Consistent with this transitional position, the geographical distribution patterns of coleopteran species recorded in this study were dominated by trans-regional distribution types, with composite categories such as North China + Northeast China, North China + Southwest China, and North China + Central China each accounting for over 50% of the total fauna. This distribution pattern—centered on the North China region but permeated by multiple adjoining biogeographic components—reflects both the reserve’s transitional status within China’s insect geographical zonation and the broader exchange between neighboring biogeographic regions. This finding is consistent with analyses of the beetle fauna of the Xiaolong Mountains, Gansu Province [54], reinforcing the general pattern of transitional faunal convergence characteristic of Coleoptera across the western Qinling Mountains.

## 5. Conclusions

This study provides the first systematic inventory of Coleoptera in the Heihe Provincial Nature Reserve, Gansu Province, recording 131 species from 108 genera and 22 families. A high proportion of monospecific genera, together with the low representation of Lampyridae, Staphylinidae, and Ptinidae alongside the dominance of Cerambycidae, most likely reflects limitations of the sampling methods used—which favored manually accessible, canopy-associated, and wood-boring taxa—rather than the reserve’s true family-level diversity. Diversity and evenness were highest in Transect II (oak-dominated mixed forest, low disturbance) and lowest in Transect IV (high human disturbance), suggesting that disturbance negatively affects community structure. Widely distributed species predominated, consistent with the reserve’s position at a Palaearctic–Oriental transition zone in the western Qinling Mountains. As a preliminary, methodologically constrained inventory, this study offers baseline data to guide future, more comprehensive surveys incorporating complementary trapping methods, multi-season sampling, and DNA barcoding, and to inform ongoing conservation monitoring in the reserve.

## Figures and Tables

**Table 1:** Characteristics of the four sampling transects in the Gansu Heihe Provincial Nature Reserve. Terrain features, dominant vegetation, and level of human disturbance are detailed for each transect.

Transect (Name)	Terrain and Hydrology	Vegetation Overview (by Elevation)	Level of Human Disturbance
I (Zhugou)	This primary gully system traverses all three management zones (experimental, buffer, core). The Heihe River flows through it, with steep rock cliffs lining its slopes (avg. width ~500 m). A 7.5 km fire access road runs along both sides.	**1500–2000 m:** Oak-coniferous and broad-leaved mixed forest, transitioning to broad-leaved mixed forest, and finally spruce coniferous forest at higher elevations. **Dominants:** *Quercus aliena var. acuteserrata*, *Fraxinus mandshurica*, *Picea crassifolia*. **Shrubs:** *Spiraea* spp. (Rosaceae). **Herbs:** Aristolochiaceae species.	1500–1800 m: High disturbance (proximity to villages). 1800–2000 m: Low-to-moderate disturbance.
II (Youcai gou)	A main tributary of the Main Gully system, originating in the experimental zone and ending at the buffer zone edge. Features a rocky streambed with steep slopes.	**500–1800 m:** Oak-dominated broad-leaved mixed forests on sunny/semi-sunny slopes and ridges. **Dominants:** *Quercus wutaishanica*, *Quercus spinosa*. **Shrubs:** *Lonicera* spp. **Herbs:** Graminoids.	High disturbance (proximity to villages and forest paths).
III (Loufangya)	Located from the buffer to the core zone; a steep, straight gully system (“First Floor” to “Dripping Cliff”) with abundant water. It converges with the Tanbanban Gully and is a key water source for the Heihe River.	**2200–2600 m:** Coniferous-broadleaved mixed forest transitioning to coniferous forest. **Dominants:** *Betula albo-sinensis*, *Betula platyphylla*, *Pinus tabuliformis*. **Feature:** Distinct bamboo understory.	Very low disturbance (remote area).
IV (Sigou)	Characterized by gentle, sunny slopes with natural Pinus tabuliformis stands at lower elevations.	**1400–2100 m:** Shallow mountain area. Pure plantations of *Pinus tabuliformis* and *P. armandii* on favorable sites; *Fraxinus* spp. in valleys. **Shrubs:** *Lespedeza* spp. **Herbs:** Graminoids.	Moderate disturbance (adjacent to Zhangjia Forest Farm and village collective forests).

**Table 2:** Inventory of Coleoptera collected from the Gansu Heihe Provincial Nature Reserve. The table lists species, family, total abundance per transect (I–IV), and global zoogeographical affinity.

Family	Genus	Species	Quantities of Coleoptera in Each Sample Line				Total	Fauna
			I	II	III	IV		
Cerambycidae	*Batocera*	*B. horsfieldi*	1	1			2	W
	*Trichoferus*	*T. campestris*	1				1	W
	*Apatophysis*	*A. sinica*	1		1		2	W
	*Ischnostrangalis*	*I. kubani*		1			1	P
	*Diboma*	*D. costata*	1				1	O
	*Corymbia*	*C. succedanea*				1	1	W
	*Tetropium*	*T. castaneumn*		1			1	W
	*Embrikstrandia*	*E. unifasciata*	1				1	W
	*Acalolepta*	*A. rusticatrix*	1				1	O
	*Anoplophora*	*A. glabripennis*	5	3	2	2	12	W
	*Psacocothes*	*P. hilari*		1			1	W
	*Vesperus*	*V. glandiforns*	1				1	P
	*Monochamus*	*M. galloprovincialis*			1		1	P
		*M. sutor*	1			1	2	P
	*Oberea*	*O. griseopennis*	2	1	3	1	7	W
	*Stenocorus*	*S. meridianus*	5	3	4	2	14	P
	*Callidium*	*C. aeneum*		1			1	O
		*C. violaceum*			1		1	O
	*Arhopalus*	*A. rusticus*	1			1	2	W
	*Olenecamptus*	*O. formosanus*	1				1	W
	*Saperda*	*S. perforata*		1			1	P
	*Macroclytus*	*M. alni*	1				1	P
	*Acanthocinus*	*A. griseus*				1	1	W
	*Mesosa*	*M. myops*			1		1	W
	*Pachyta*	*P. quadrimaculata*	1			1	2	W
	*Agapanthia*	*A. amurensis*		1		1	2	W
	*Judolia*	*J. judia*		1			1	P
Oedemeridae	*Oedemeronia*	*O. virescens*	1		1	1	3	P
	*Nacerdes*	*N. melanura*	2	1	1	1	5	O
	*Nacerdes*	*N. waterhousei*	7	3	4	4	18	W
	*Chrysanthia*	*C. genicnlatachinensis*		1			1	W
Lucanidae	*Prosopocoilus*	*P. astacoides*	4	6	3	5	18	W
	*Prismognathus*	*P. dauricus*	1	1	2		4	P
	*Lucanus*	*L. hermani*			1	1	2	W
Melolonthidae	*Melolontha*	*M. hippocastani*		1			1	W
		*M. melolontha*	2	2	1	1	6	P
	*Amphimallon*	*A. solstitialis*	12	8	6	9	35	P
	*Holotrichia*	*H. oblita*	11	5	7	8	31	W
		*H. lata*				1	1	O
		*H. picea*			1		1	O
	*Polyphylla*	*P. laticollis*	4	2	6	2	14	W
	*Exolontha*	*E. serrulata*	1		1		2	O
Scarabaeidae	*Stenocara*	*S. orientalis*	13	7	12	7	39	W
Cetoniidae	*Neophaedimus*	*N. auzouxi*		1	1		2	W
	*Cetonia*	*C.aurata*	1				1	O
	*Protaetia*	*P. orientalis*	1				1	O
		*P. brevitarsis*	1				1	W
		*P. pryeri*				1	1	P
	*Clinterocera*	*C. mandarina*	1				1	W
	*Cosmiomorpha*	*C. setulosa*				1	1	O
	*Dicranocephalus*	*D. adamsi*		1		1	2	W
Rutelinae	*Phyllopertha*	*P. horticola*	2	5	2	2	11	W
	*Callistethus*	*C. plgiicollis*	3	1	1		5	W
	*Adoretus*	*A. sinicus*		1	1	1	3	W
	*Mimela*	*M. splendens*	3	5	2	2	12	W
Geotrupidae	*Enoplotrupes*	*E. sinensis*				1	1	O
Silphidae	*Necrodes*	*N. littoralis*		1		1	2	O
	*Nicrophorus*	*N. nepalensis*	1	1			2	P
		*N. japonicus*	1				1	W
	*Calathus*	*C. halensis*	15	7	13	10	45	W
Cantharidae	*Themus*	*T. imperialis*	13	6	11	16	46	W
		*T. stigmaticus*	1	2			3	O
		*T. kaszabi*	3	2	1	2	8	O
	*Lycocerus*	*L. hamatus*		1			1	W
	*Cantharis*	*C. rufa*	5	10	1	1	17	P
Lampyridae	*Pyrocoelia*	*P. rufa*		1			1	O
Lycidae	*Lycostomus*	*L. porphyrophorus*	2	1	1	1	5	O
		*L. similis*	1	1	1		3	O
Coccinellidae	*Harmonia*	*H. axyridis*	18	16	5	2	41	W
	*Adalia*	*A. bipunctata*	1	1			2	W
	*Vibidia*	*V. duodecimguttata*	7	9	5	2	23	W
	*Halyzia*	*H. sedecimguttata*	9	4	2	3	18	P
	*Coccinella*	*C. longifasciata*		1			1	W
		*C. transversoguttata*	3	5	3	4	15	W
	*Oenopia*	*O. scalaris*	2	1	2	1	6	W
	*Illeis*	*I. koebelei*		1	1		2	W
	*Halyzia*	*H. hauseri*				1	1	W
	*Lemnia*	*L. saucia*				1	1	W
	*Aiolocaria*	*A. hexaspilota*	3	1	1		5	W
	*Calvia*	*C. quatuordecimpunctata*			1		1	W
		*C. championorum*		1	1		2	W
	*Epilachna*	*E. plicata*	23	15	12	10	60	W
Carabidae	*Amara*	*A. brevicollis*		1	1		2	W
	*Catascopus*	*C. smaragdulus*	1	2	1	1	5	W
	*Harpalus*	*H. rufipes*	2	3	1	3	9	W
		*H. sinicus*	6	5	1	1	13	W
		*H. griseus*			1		1	W
	*Chlaenius*	*C. inops*	2	1			3	W
	*Cymindis*	*C. daimio*			2	2	4	W
		*C. lacon*	1				1	P
	*Dolichus*	*D. halensis*	2	1	1	1	5	W
	*Anisodactylus*	*A.binotatus*	3	4	2	3	12	W
	*Platynus*	*P. magnus*	1		1	1	3	W
	*Pheropsophus*	*P. jessoensis*			1		1	W
Curculionidae	*Curculio*	*C. dentipes*	5	6	3	2	16	W
	*Hylobius*	*H. harotdi*	1	1	1	1	4	W
	*Sphenophorus*	*S. venatus*	1		1	1	3	W
	*Eucryptorrhynchus*	*E. scrobiculatus*	1			1	2	W
	*Dermatoxenus*	*D. caesicollis*		2	1		3	W
	*Cyrtepistomus*	*C. castaneus*		1	1		2	W
	*Lixus*	*L. ochreceus*	1	3			4	W
	*Sympiezomias*	*S. citri*	1				1	O
	*Anthonomus*	*A. quadrigibbus*	5	1		1	7	P
	*Calomycterus*	*C. obconicus*	1	2	1	1	5	O
Elateridae	*Agriotes*	*A. fuscicolis*	1	1		1	3	W
	*Tetrigus*	*T. lewisi*	2	3	2	3	10	W
	*Agrypnus*	*A. argillaceus*		1		1	2	W
Tenebrionidae	*Lagria*	*L. hirta*	12	3	5	4	24	W
		*L. nigricollis*	17	12	12	15	56	W
		*L. scutellaris*	6	8	12	5	31	O
		*L. ventralis*	1	1	1		3	O
Chrysomelidae	*Diabrotica*	*D. virgifera*	2	1	3	1	7	P
		*D. barberi*	3	3	4	3	13	O
	*Aulacophora*	*A. lewisii*	18	15	5	4	42	W
		*A. indica*	1				1	W
	*Plagiodera*	*P. versicolora*	1	1	1	1	4	W
	*Clytra*	*C. quadripunctata*	2		1		3	P
	*Pyrrhalta*	*P. aenescens*	4	3	1	2	10	P
	*Cryptocephalus*	*C. hyacinthinus*			1		1	O
		*C. festivus* Jacoby	1				1	O
	*Galerucella*	*G. grisescens*	2	2	1	1	6	W
	*Chrysolina*	*C. aurichalcea*	1	3		1	5	W
	*Altica*	*A. birmanensis*	2	2	2	2	8	O
	*Lema*	*L. decempunctata*	2	1		2	5	W
Meloidae	*Epicauta*	*E. hirticornis*			1		1	W
	*Lytta*	*L. stygica*	1			1	2	W
Buprestidae	*Agrilus*	*A. planipennis*	2	1	1	2	6	W
		*A. pseudonarrowi*		1			1	W
	*Chrysobothris*	*C. affinis*	1				1	W
Staphylinidae	*Paederus*	*P. fuscipes*			1	2	3	O
Anobiidae	*Stegobium*	*S. paniceum*	1		1		2	W

Note: P. Palaearctic species; O. Oriental species; W. Cosmopolitan species.

**Table 3:** Distribution of Coleoptera species across Chinese zoogeographical regions in the Gansu Heihe Provincial Nature Reserve. Patterns are listed with species counts and their corresponding percentage of the total fauna.

Serial Number	Zoogeographical Distribution in China	Number of Species	Proportion
1	North China Region	99	75.57%
2	North China Region + Northeast Region	71	54.20%
3	North China Region + Inner Mongolia − Xinjiang Region	43	32.82%
4	North China Region + Qinghai − Tibet Region	37	28.24%
5	North China Region + Southwest Region	68	51.91%
6	North China Region + Central China Region	69	52.67%
7	North China Region + South China Region	55	41.98%
8	North China Region + Northeast Region + Inner Mongolia − Xinjiang Region	37	28.24%
9	North China Region + Northeast Region + Qinghai − Tibet Region	31	23.66%
10	North China Region + Northeast Region + Southwest Region	47	35.88%
11	North China Region + Northeast Region + Central China Region	52	39.69%
12	North China Region + Northeast Region + South China Region	41	31.30%
13	North China Region + Inner Mongolia − Xinjiang Region + Qinghai − Tibet Region	25	19.08%
14	North China Region + Inner Mongolia − Xinjiang Region + Southwest Region	29	22.14%
15	North China Region + Inner Mongolia − Xinjiang Region + Central China Region	28	21.37%
16	North China Region + Inner Mongolia − Xinjiang Region + South China Region	23	17.56%
17	North China Region + Qinghai − Tibet Region + Southwest Region	30	22.90%
18	North China Region + Qinghai − Tibet Region + Central China Region	26	19.85%
19	North China Region + Qinghai − Tibet Region + South China Region	23	17.56%
20	North China Region + Southwest Region + Central China Region	55	41.98%
21	North China Region + Southwest Region + South China Region	47	35.88%
22	North China Region + Northeast Region + Inner Mongolia − Xinjiang Region + Qinghai − Tibet Region	22	16.79%
23	North China Region + Northeast Region + Inner Mongolia − Xinjiang Region + Southwest Region	24	18.32%
24	North China Region + Northeast Region + Inner Mongolia − Xinjiang Region + Central China Region	27	20.61%
25	North China Region + Northeast Region + Inner Mongolia − Xinjiang Region + South China Region	22	16.79%
26	North China Region + Inner Mongolia − Xinjiang Region + Qinghai − Tibet Region + Southwest Region	19	14.50%
27	North China Region + Inner Mongolia − Xinjiang Region + Qinghai − Tibet Region + Central China Region	15	11.45%
28	North China Region + Inner Mongolia − Xinjiang Region + Qinghai − Tibet Region + South China Region	13	9.92%
29	North China Region + Qinghai − Tibet Region + Southwest Region + Central China Region	25	19.08%
30	North China Region + Qinghai − Tibet Region + Southwest Region + South China Region	23	17.56%
31	North China Region + Southwest Region + Central China Region + South China Region	46	35.11%
32	North China Region + Northeast Region + Inner Mongolia − Xinjiang Region + Qinghai − Tibet Region + Southwest Region	16	12.21%
33	North China Region + Northeast Region + Inner Mongolia − Xinjiang Region + Qinghai − Tibet Region + Central China Region	14	10.69%
34	North China Region + Northeast Region + Inner Mongolia − Xinjiang Region + Qinghai − Tibet Region + South China Region	12	9.16%
35	North China Region + Inner Mongolia − Xinjiang Region + Qinghai − Tibet Region + Southwest Region + Central China Region	14	10.69%
36	North China Region + Inner Mongolia − Xinjiang Region + Qinghai − Tibet Region + Southwest Region + South China Region	13	9.92%
37	North China Region + Qinghai − Tibet Region + Southwest Region + Central China Region + South China Region	23	17.56%
38	North China Region + Northeast Region + Inner Mongolia − Xinjiang Region + Qinghai − Tibet Region + Southwest Region + Central China Region	11	8.40%
39	North China Region + Northeast Region + Inner Mongolia − Xinjiang Region + Qinghai − Tibet Region + Southwest Region + South China Region	12	9.16%
40	North China Region + Inner Mongolia − Xinjiang Region + Qinghai − Tibet Region + Southwest Region + Central China Region + South China Region	13	9.92%
41	North China Region + Northeast Region + Inner Mongolia − Xinjiang Region + Qinghai − Tibet Region + Southwest Region + Central China Region + South China Region	12	9.16%

**Table 4:** *α*-Diversity indices of coleopteran communities across the four sampling transects (I–IV) in the Gansu Heihe Provincial Nature Reserve.

Line	Genus	Species	Individual	Diversity Indices				(D) Dominance Index	(J) Evenness Index	(R) Species Richness
				H′ (F)	H′ (S)	H′ (S)	H′ (FGS)			
Transect I	78	87	319	2.5183	3.7176	3.9166	10.1525	0.0721	0.8770	14.9171
Transect II	71	82	249	2.5081	3.7556	3.9602	10.2239	0.0643	0.8987	14.6807
Transect III	65	75	202	2.5721	3.6006	3.8564	10.0291	0.0644	0.8932	13.9405
Transect IV	66	72	185	2.6578	3.6388	3.8333	10.1298	0.0865	0.8963	13.6006

**Table 5:** Similarity of coleopteran communities among the four sampling transects. Values above the diagonal indicate the number of shared species; values below the diagonal are Jaccard similarity coefficients.

Transect	I	II	III	IV
I		59	52	56
II	0.52		56	52
III	0.46	0.55		51
IV	0.53	0.50	0.53	

## Data Availability

All data generated or analyzed during this study are all included in this published article. All of the raw data can be obtained publicly.
